# Supplementary material for: Intracellular Staphylococcus aureus infection in human osteoblasts: circRNA expression analysis
Source: Heliyon. 2024 Mar 21;10(7):e28461. doi: 10.1016/j.heliyon.2024.e28461 (PMC10979106; doi:10.1016/j.heliyon.2024.e28461)
Supplement: Multimedia component 1 [file mmc1.docx]

**Intracellular *Staphylococcus aureus* infection in human osteoblasts: circRNA expression analysis**

Liubing Li^1△^, Min Wang^2△^, Qi Chen^2△^, Mingxing Zhang^1,3△^, Zhihao Chen^1^, Mingxiao Han^2^, Chenhao Zhao^2^, Zonggang Xie^1^, Qirong Dong^1*^, Haifang Zhang^2*^

1. Department of Orthopedics, The Second Affiliated Hospital of Soochow University, Suzhou, China
2. Department of Clinical Laboratory, The Second Affiliated Hospital of Soochow University, Suzhou, China
3. Department of Orthopedics, Shuofang Hospital of Xinwu District, Wuxi, China

△These [author](C:/Users/perfectguo/AppData/Local/youdao/dict/Application/8.9.3.0/resultui/html/index.html#/javascript:;)s contributed equally to this work.

*Corresponding author: Haifang [Zhang (haifangzhang@suda.edu.cn)](mailto:Zhang（haifangzhang@sina.com）;Hong); Qirong Dong (dongqirong@suda.edu.cn).

**Abstract**

*Staphylococcus aureus* (*S. aureus*) has the ability to invade human cortical bones and cause intracellular infections in osteoblasts, which may lead to a long-term infection that is difficult to eliminate. It is critical to identify the underlying mechanisms of the osteoblast response to the intracellular *S. aureus*. More recently, multiple circular RNA (circRNA) functions have been identified, including serving as protein scaffolds or miRNA sponges and being translated into polypeptides. The role that circRNAs play in intracellular *S. aureus* infection of osteoblasts has not, to our knowledge, been investigated. Here, we established an intracellular infection model of *S. aureus* in osteoblasts and compared the circRNA expression of osteoblasts between the infected and control groups using RNA sequencing technology, by which a significant difference was found. In total, 117 upregulated and 125 down-regulated differentially expressed circRNAs (DEcircRNAs) were identified, and reverse transcription-quantitative PCR was employed to validate the results of RNA sequencing. Gene Ontology and Kyoto Encyclopedia of Genes and Genomes pathway analyses demonstrated that DEcircRNAs were enriched in processes associated with macromolecule modification, cellular component organization or biogenesis, and intracellular non-membrane-bound organelles. Finally, a potentially important network of circRNA-miRNA-mRNA based on the DEcircRNAs was constructed. Overall, this study revealed the circRNA expression profile of human osteoblasts infected by intracellular *S. aureus* for the first time, and identified the circRNAs that may contribute to the pathogenesis of infectious diseases caused by intracellular *S. aureus* infection in human osteoblasts.

**Keywords**: osteoblast, intracellular infection, *Staphylococcus aureus*, circRNAs, orthopedic infection

1. **Introduction**

*Staphylococcus aureus* (*S. aureus*) is a Gram-positive pathogen known for causing infections in almost all human organs, including bones and joints. While *S. aureus* was originally considered to be an extracellular infection pathogen, an increasing number of studies have reported that it also has the capacity of intracellular infection [1, 2]. *S. aureus* may escape the phagocytosis of phagosomes, adapting to the adverse intracellular environment [3]. Host cells were used as carriers and *S. aureus* survived intracellularly for several days [4]. Once in the adaptive environment, *S. aureus* cells can destroy the host cells and infect other host cells [5]. *S. aureus*, which can cause intracellular infection, is more toxic and more likely to cause serious systematic infections in humans than the ones that lead to extracellular infection [6, 7]. Furthermore, intracellular *S. aureus* spreads infection and is protected from antibiotics [8].

Severe bone tissue infection causes progressive inflammatory destruction of the bone, and *S. aureus* is the most common pathogen. The established clinical approach for chronic osteomyelitis treatment involves debridement of the infected bone, tissue reconstruction, and administration of long-term antibiotics. Due to the poor local blood supply of chronic osteomyelitis lesions and dead bone formation, it is difficult for antibiotics to attain an effective bactericidal concentration in the lesion, with a poor treatment effect and considerable side effects. The direct use of sensitive antibiotics in the local lesion has high performance requirements of the selected antibiotic carrier. This carrier must not only ensure a sufficient antibiotic load but also enable continuous and stable antibiotic releases to completely eliminate the bacterial biofilm on the surface of dead bone and scar tissue. The current treatment methods for osteomyelitis are often traumatic, costly, and frequently lead to further selection of *S. aureus* antibiotic-resistant strains[9]. The increasing incidence of antibiotic-resistant *S. aureus* strains could explain the recurrent episodes of osteomyelitis in treated patients. Orthopedic infections by *S. aureus* can relapse even after apparent clearance of the initial infection [10]. It has been shown that bacteria causing infection can enter and reside within osteoblasts [10]. The intracellular bacteria proliferate and colonize adjacent areas forming biofilm aggregates, and these populations of intracellular bacteria and biofilms present a complicated clinical problem [10]. It was reported that *S. aureus* internalization into osteoblasts plays a critical role in the persistence and recurrence of osteomyelitis, and the EGFR/FAK and c-Src signaling pathways mediate osteoblastic *S. aureus* internalization[11] .

Circular RNA (circRNA) is an emerging class of non-coding RNA molecules, which is present largely in eukaryotic cells. The expression of circRNA is tissue- and disease-specific, and affects disease development. In recent years, many reports have demonstrated the importance of circRNAs in pathophysiology in that they can function as microRNA (miRNA) sponges to regulate transcription and gene expression, some of which can even be translated into proteins [12-17]. CircRNAs are involved in the pathogenesis of various diseases, with high sequence conservation, structural stability, and abundance and having certain tissue and disease specificities [18], making them of considerable interest in the field of biology. They also have potential value in disease diagnosis and as prognostic biomarkers and therapeutic targets [19, 20].

The involvement of circRNAs in the response of osteoblasts to intracellular *S. aureus* infection remains largely uninvestigated. In this study, we investigated the circRNA expression profile of human osteoblasts infected by intracellular *S. aureus*, and analyzed circRNAs which may contribute to the development of orthopedic infectious diseases caused by human osteoblastic intracellular *S. aureus* infection.

1. **Methods and Materials**
   1. **Osteoblast culture**

Human osteoblast Hfob1.19 cells were purchased from the Cell Library of Shanghai, Chinese Academy of Sciences. Hfob1.19 cells were cultured in DMEM and F-12 medium (1:1) containing 15% fetal bovine serum and 300 g/ml G418 at 37°C under 5% CO_2_. Histochemical staining for alkaline phosphatase was applied to investigate the functional activity of the osteoblasts.

- 1. **Bacterial strains and growth conditions**

1. *aureus* strain (ATCC25923) used in this study was, stored in the clinical microbiology laboratory. To resuscitation the bacteria, *S*. *aureus* was culture on the blood plate first. Then, one colony was choosed and transferred into 1 mL Luria-Bertani (LB) isotonic liquid medium by the inoculating loop and shaked at 37°C and 250 rpm overnight. Next day, 200 µL the overnight cultured bacterial solution was added to 20 mL LB medium and shaken at 37°C and 250 rpm until the OD_600_ reached at 0.6.

**2.3 CFSE fluorescent labeling**

Before the invasion assay, 1 × 10^9^/ml *S*. *aureus* cells were suspended in 1 μg/ml carboxyfluorescein succinimidyl ester (CFSE) and shaken for 30 min at 37°C and 250 rpm for fluorescent labeling.

**2.4 Invasion assay**

Hfob 1.19 cells were seeded in a six-well plate at 1 × 10^6^ cells per well, and once cell fusion was achieved (6–8 days), Hfob 1.19 cells were infected with *S. aureus* from multiplicity of infection (MOI) 25:1 to 500:1. Before the collection of post-infection cells, gentamicin (200 µg/mL) was added into the culture to eliminate the extracellular bacteria. Then, at 30 min and 1, 2, 4, and 8 h post infection, cells were collected for further analysis after washed three times with phosphate-buffered saline. *S. aureus* intracellular infection in osteoblasts was detected by flow cytometry.

**2.5 Preparation of total RNA and RNA sequencing of osteoblasts after *S*. *aureus* infection**

Both infected and control cells were extracted total RNA by TRIzol reagent (Invitrogen, USA) .The quality and concentration of RNA extracted was assessed by agarose electrophoresis and NanoDrop-1000 (Thermo Fisher, USA) respectively. RNA samples were then sent to Cloudseq Biotech Inc. (Shanghai, China) for sequencing. The cDNA libraries were quantified by BioAnalyzer 2100 system (Agilent Technologies, USA), and finally sequenced on an Illumina HiSeq Sequencer NovaSeq 6000.

**2.6 Identification of differentially expressed circRNAs (DEcircRNAs)**

Based on the above RNA sequencing data, circRNAs were detected by DCC software (v0.4.4) and identified by the circBase database and the circ2Trait circular RNA-disease database. The differentially expressed circRNAs (DEcircRNAs) were analyzed by edgeR software (v3.16.5). The DEcircRNA was calculated between two or two sets of samples. Calculate the fold change (fold change) and P-value. The fold change> =2.0 and P-value <0.05 were used as the threshold for DEcircRNAs.

**2.7 Verification of circRNA expression accuracy by RT-qPCR**

The verification of circRNA sequencing data was performed by reverse transcription-quantitative PCR (RT-qPCR). Six DEcircRNAs with good potential were randomly screened out, three of them were high expressed and three of them were low expressed. The designed specific primers for these DEcircRNAs were shown in Table S1. The expression level of *GAPDH* was taken as an internal standard, and the relative expression levels of randomly selected DEcircRNAs were calculated by the 2^−ΔΔCt^ method. The experiments were performed in triplicate.

**2.8** **Functional analysis of DEcircRNAs**

Gene Ontology (GO) annotation including Molecular Function (MF), Biological Process (BP) and Cell Component (CC) was used to analysis the functions of DEcircRNAs. The GO terms with a P-value <0.05 were considered statistically significant. Then we analyzed the potential pathways associated with by Kyoto Encyclopedia of Genes and Genomes (KEGG) pathway analysis. P-value <0.05 was used as the threshold for significant enrichment.

**2.9 Construction of the circRNA-miRNA interaction network**

CircRNA regulates mRNA expression through absorbing miRNA, which functions as a miRNA sponge. Targetscan and miRanda software were used to predict potential miRNAs targetting to DEcircRNAs. From the list of predicted circRNA-miRNA, we used Cytoscape software to analyze circRNA-miRNA interaction and generate a network diagram which may be associated with the function of DEcircRNA.

**2.10 Statistical analysis**

Differences between the two groups were analyzed for statistical significance by the Student’s *t*-test. *P* < 0.05 indicated statistical significance.

**2.11 Data availability**

The raw RNA-seq data can be available by the accession number of GSE217234 in GEO database.

1. **Results**

**3.1 Development of an *S. aureus* intracellular infection model in osteoblasts**

In the invasion experiment, the infection dose of MOI was from 25:1 to 500:1 and the infection duration was from 30 min to 8 h. Two indicators of bacterial invasion were assessed: one was the invasion rate, which was the percentage of osteoblasts containing CFSE-labeled *S. aureus* among all osteoblasts, and the other was the average CFSE fluorescence intensity in osteoblasts. As the infection dose increased from an MOI of 25:1 to 500:1, the invasion rate increased from 13.58% to 97.28% after human osteoblast Hfob1.19 cells were infected with *S. aureus* after 2 h (Fig. 1A), and the correspondent average CFSE fluorescence intensity in osteoblasts increased significantly (Fig. 1B). However, when the MOI ranged from 250 to 500, there was only a slight increase in the bacterial invasion rate. Therefore, the MOI was fixed at 250:1 in this study.

The invasion rate and mean fluorescence intensity in invading osteoblasts with the different bacterial invasion times from 30 min to 8 h with the MOI of 250 were shown in Figure 2A and Figure 2B, respectively. With a 30-min infection time, the bacterial invasion rate was 4.78%. When the infection time was 4 h, the bacterial invasion rate of osteoblasts reached the highest point of 51.36%, and then decreased with a longer infection time, falling to 31.65% at 8 h.

Our study revealed that cell survival remained stable between 30 min and 4 h, with a gradually increasing number of Hfob1.19 osteoblasts containing bacteria at the infection doses ranging from a MOI of 25 to 500 and reaching 93.28% by an MOI of 250:1. Therefore, samples with a 4-h infection time and an MOI of 250:1 were suitable for library construction and sequencing. Finally, the infection model of *S. aureus* ATCC25923 interacted with the Hfob1.19 osteoblasts at an infection dose of MOI of 250:1 for 4 h was established.

**3.2 Identification of the DEcircRNAs of osteoblasts on *S. aureus* intracellular infection**

We detected a total expression of 8759 circRNAs, and the length of most circRNAs was < 2000 nt (Fig. 3). Furthermore, we classified all the identified circRNAs based on the different locations on the chromosomes. The results showed that these circRNAs were widely distributed across all chromosomes, and among them 832 were located on Chr1 but only 14 were located on ChrY (Fig. 4). Interestingly, among the 8759 identified circRNAs, 62.6% were exonic circRNAs, 15.9% were intronic circRNAs, and only 0.5% of the circRNAs were intergenic (Fig. 5).

Using the normalized number of reads, the differentially expressed circRNAs between the infected and control groups were calculated. Multiple changes and the *P*-value were calculated. In the screening analysis with a fold change ≥ 2.0 and *P*-value <0.05 as the threshold for differential circRNAs, 242 circRNAs obtained from the test samples and control were essentially differentially expressed, with 117 upregulated and 125 downregulated. The top ten circRNAs upregulated and downregulated were listed in Table 1 and Table 2, respectively.

**3.3 Verification of differential circRNA gene expression by RT-qPCR**

We randomly selected three DEcircRNAs upregulated and downregulated, respectively, for further verification by RT-qPCR: chr1:213251038-213290752+, chr17:44143903-44172067-,chr8:141856359-141889736-, chr2:40655613-40657441-, chr1:180953813-180962561-, chr8:141874411-141900868-.

As shown in Figure 6, the expression of circRNAs chr17:44143903-44172067-, chr8:141856359-141889736-, and chr1:213251038-213290752+ was elevated in infected osteoblasts. By contrast, chr8:1418741411-141900868-, chr2:40655613-40657441-, and chr1:180953813-180962561- were downregulated in infected osteoblasts. These results were consistent with the RNA sequencing data, and these six circRNAs were stable in normal osteoblasts infected by *S. aureus*, suggesting that they may serve as reliable biomarkers.

**3.4 Functional analysis of DEcircRNAs**

GO annotation divides the differently expressed genes into three categories: biological process (BP), cell component (CC), and molecular function (MF). For DEcircRNAs upregulated in the *S*. *aureus* infection group, the three most important GO processes in the BP subgroup were macromolecule modification, protein modification process, and cellular protein modification process (Fig. 7A). Cellular component organization, cellular component organization or biogenesis, and cellular process were the top three processes in the BP subgroup regarding downregulated DEcircRNAs (Fig. 7B). In the CC subgroup, the most enriched GO terms in upregulated DEcircRNAs were nucleoplasm part, extrinsic component of plasma membrane, and extrinsic component of membrane (Fig. 7C), whereas, intracellular, intracellular part, and intracellular non-membrane-bourded organelle were the most significant GO terms regarding the downregulated DEcircRNAs (Fig. 7D). The top three GO processes included catalytic activity, nucleotide binding, and nucleoside phosphate binding in the MF subgroup among the upregulated DEcircRNAs (Fig. 7E). Heterocyclic compound binding, poly(A) RNA binding, and ATP binding were the top three processes in the MF subgroup regarding the downregulated DEcircRNAs (Fig. 7F).

In KEGG pathway enrichment analysis, chemokine signaling pathway, bacterial invasion of epithelial cells, and regulation of actin cytoskeleton were the most significant pathways enriched among the host genes of the upregulated DEcircRNAs (Fig. 8A). Herpes simplex infection, endocytosis, and chemokine signaling pathway were the most significantly enriched pathways of the downregulated DEcircRNAs (Fig. 8B).

**3.5 Analysis of the circRNA-miRNA interaction networks**

hsa_circ_0085769 was highly expressed in *S. aureus*-infected osteoblasts, and its expression was positively correlated with PTK2 expression at the mRNA level based on the RNA sequencing results of this study. According to the bioinformatic prediction, hsa_circ_0085769 was found as available as the major miRNA-target site for hsa-miR-6886-5p, hsa-miR-6812-3p, hsa-miR-6769b-5p, hsa-miR-6765-5p, and hsa-miR-182-5p, of which hsa-miR-6886-5p, hsa-miR-6765-5p, and hsa-miR-182-5p can also bind hsa_circ_0002483, which was significantly downregulated in *S. aureus*-infected osteoblasts compared to the control in this study (Fig.9). Interestingly, hsa_circ_0002483 expression was negatively correlated with PTK2 in this study. Because PTK2 participates in the bacterial invasion pathway, hsa_circ_0085769 and hsa_circ_0002483 are probably associated with the osteogenic infection caused by *S. aureus* via interaction with their common miRNAs.

1. **Discussion**

Osteocytes are the most abundant of all cell types that constitute and maintain bone, being of the osteoblast lineage derived from BMSC proliferation and differentiation. However, because they are located deep in the cortical bone, little attention has been paid to the role of osteocytes in osteomyelitis. Yang *et al* showed that *S. aureus* is able to infiltrate both occupied and unoccupied osteocyte lacunae in cultured human bone in vitro when observed by electron microscopy, and methicillin-resistant *S. aureus* was internalized by osteocyte-like cells when co-cultured *in vitro* [21, 22]. Relapsing osteomyelitis decades after the initial infection suggests that *S. aureus* can be present in human osteocytes [23]. It remains for decades and can persist over most of the lifespan of an organism. The concern is that intracellular infection of osteoblasts in this way can convert a brief bacterial infection into a lasting source of infection. Ji *et al*. raised the potential of targeting EGFR/FAK and c-Src of osteoblasts as adjunctive therapeutics for treating chronic *S. aureus* osteomyelitis [11]. Indeed, the difficulty of treating bone and joint infection compared with the soft tissue stems from the inherent physiological and anatomical differences of bone and joints, which makes it more difficult for some commonly used antibiotics to penetrate and achieve sufficient concentration to achieve a bactericidal effect [24] .

CircRNAs are noncoding RNAs, which may provide novel insights into the pathology of osteomyelitis. However, little is known about the roles of circRNAs in the pathogenesis of osteomyelitis, particularly in osteoblasts. Here, we studied the intracellular infection process of *S*. *aureus* and analyzed the circRNA expression profile of *S*. *aureus*-infected osteoblasts. In our study, an intracellular survival model of *S. aureus*-infected osteoblasts was established. In previous quantitative studies of *S. aureus* invasion into cells, infected cells were first chemically cracked, and the bacteria released extracellularly after lysis were collected [25]. The collected bacteria were inoculated into bacterial solid culture plates and grown overnight by serial concentration dilution methods, and bacterial colonies were counted the next day to yield the average number of bacteria that had invaded per cell [26]. However, this method can only count the bacteria that survive the lysis, and not those that die after invading the cells, making it difficult to accurately assess the actual number of invading bacteria. In addition, when calculating the number of bacteria contained in cells, the method assumes that bacteria invade all cells simultaneously, thus it cannot accurately reflect the changing process of bacterial invasion over time with the increase of infection dose and time. In our study, we developed a method to simultaneously detect invading bacteria and those that die, thus the actual number of bacteria invading the cells can be determined.

In this study, we applied high-throughput sequencing to analyze intracellular *S*. *aureus*-infected osteoblasts and found 117 upregulated and 125 downregulated DEcircRNAs. The top ten circRNAs upregulated in osteoblasts during infection by *S. aureus* have not been reported before*,* except hsa_circ_0001777. It has been found that hsa_circ_0001777 might act as a miRNA sponge for miR-182 in triple-negative breast cancer [27]. Among the top ten downregulated circRNAs, several have been reported, such as, hsa_circ_0005232, involved in the pathogenesis of aseptic loosening after total hip arthroplasty [28], and hsa_circ_0007905, associated with cervical squamous cell carcinoma [29]. The functions and pathways of the DE circRNAs involved in osteoblasts *S. aureus* infection require further research.

Numerous studies have shown that circRNAs plays an important role in osteoblast evolution, differentiation, and functional expression. For example, Dou *et al*. analyzed differential expression of the entire transcriptome, including circRNAs, lncRNAs, miRNAs, and mRNAs, during osteoclast formation and found that hsa_circ_0007873 upregulated miR-103 expression in the constituted circRNA-miRNA interaction network, whereas hsa_circ_0010763 and hsa_circ_0015622 downregulated miR-103 expression [30]. The expression of miR-103 has been shown to be associated with the inhibition of osteoblast proliferation [31]. The present study revealed that hsa_circ_0085769 can be used as the major target site for miRNA. Because hsa_circ_0085769 expression was upregulated in *S. aureus* intracellular-infected osteoblasts, and was positively correlated with PTK2 protein expression, it may play a positive regulatory role in epithelial cell signaling upon bacterial invasion by binding to hsa-miR-6886-5p. Nicola *et al* performed an in-depth analysis of the effect of long-term *S. aureus* infection on the transcriptional program of human osteoblast-like cells. Integration of energy metabolism and signaling by receptor tyrosine kinase (PTK2) categories were identified [32]. The hsa_circ_0002483 can also be used as a major miRNA-target site for hsa-miR-6886-5p. Because hsa_circ_0002483 is clearly differentially expressed in *S. aureus*-infected osteoblasts, and its expression level is negatively correlated with PTK2 protein expression levels, hsa_circ_0002483 may negatively regulate epithelial cell signaling upon bacterial invasion by binding to hsa-miR-6886-5p, and subsequently affect the osteoblasts during infection by *S. aureus*. At the same time, circRNA-protein interactions received ever greater attention, and the circRNAs may interact with some proteins in the nucleus to regulate the occurrence and recurrence processes of intracellular infection.

This study is purely descriptive; therefore, it is premature to come to a conclusion and we simply provide our data, but we feel that this should be recognized. However, we emphasize the need for further investigation into the specific functions of these circRNAs and the mechanisms by which they regulate intracellular infection of osteoblasts. In addition, these circRNAs will be validated in clinical samples in our next research plan.

**Funding**

This study was supported by the Suzhou Health Talents Program (2020092), Gusu Health Youth Talent of Suzhou (GSWS2019039, GSWS2020030), the Science and Technology Program of Suzhou (SKY2021007), Discipline Construction of The Second Affiliated Hospital of Soochow University (XKTJ-TD202001), Academic Research Fund for College Students of Soochow University (KY2022099013), and Academic Research Fund for College Students of Soochow Medical College (2022YXYKWKY065).

**Conflict of interests**

The authors declare no conflict of interests.

**References**

[1] K.D. Alder, I. Lee, A.M. Munger, H.K. Kwon, M.T. Morris, S.V. Cahill, et al., Intracellular Staphylococcus aureus in bone and joint infections: A mechanism of disease recurrence, inflammation, and bone and cartilage destruction, Bone. 141 (2020) 115568.

[2] D. Yang, A.R. Wijenayaka, L.B. Solomon, S.M. Pederson, D.M. Findlay, S.P. Kidd, et al., Novel Insights into Staphylococcus aureus Deep Bone Infections: the Involvement of Osteocytes, mBio. 9 (2) (2018)

[3] M. Wang, Z. Fan and H. Han, Autophagy in Staphylococcus aureus Infection, Frontiers in cellular and infection microbiology. 11 (2021) 750222.

[4] K. Stelzner, A.C. Winkler, C. Liang, A. Boyny, C.P. Ade, T. Dandekar, et al., Intracellular Staphylococcus aureus Perturbs the Host Cell Ca(2+) Homeostasis To Promote Cell Death, mBio. 11 (6) (2020)

[5] M. Kubica, K. Guzik, J. Koziel, M. Zarebski, W. Richter, B. Gajkowska, et al., A potential new pathway for Staphylococcus aureus dissemination: the silent survival of S. aureus phagocytosed by human monocyte-derived macrophages, PloS one. 3 (1) (2008) e1409.

[6] S. Clement, P. Vaudaux, P. Francois, J. Schrenzel, E. Huggler, S. Kampf, et al., Evidence of an intracellular reservoir in the nasal mucosa of patients with recurrent Staphylococcus aureus rhinosinusitis, The Journal of infectious diseases. 192 (6) (2005) 1023-1028.

[7] P. Sendi and R.A. Proctor, Staphylococcus aureus as an intracellular pathogen: the role of small colony variants, Trends in microbiology. 17 (2) (2009) 54-58.

[8] S.M. Lehar, T. Pillow, M. Xu, L. Staben, K.K. Kajihara, R. Vandlen, et al., Novel antibody-antibiotic conjugate eliminates intracellular S. aureus, Nature. 527 (7578) (2015) 323-328.

[9] H.W. Boucher, G.H. Talbot, J.S. Bradley, J.E. Edwards, D. Gilbert, L.B. Rice, et al., Bad bugs, no drugs: no ESKAPE! An update from the Infectious Diseases Society of America, Clinical infectious diseases : an official publication of the Infectious Diseases Society of America. 48 (1) (2009) 1-12.

[10] D.H. Dusane, D. Kyrouac, I. Petersen, L. Bushrow, J.H. Calhoun, J.F. Granger, et al., Targeting intracellular Staphylococcus aureus to lower recurrence of orthopaedic infection, Journal of orthopaedic research : official publication of the Orthopaedic Research Society. 36 (4) (2018) 1086-1092.

[11] Z. Ji, J. Su, Y. Hou, Z. Yao, B. Yu and X. Zhang, EGFR/FAK and c-Src signalling pathways mediate the internalisation of Staphylococcus aureus by osteoblasts, Cellular microbiology. 22 (10) (2020) e13240.

[12] D. Han, J. Li, H. Wang, X. Su, J. Hou, Y. Gu, et al., Circular RNA circMTO1 acts as the sponge of microRNA-9 to suppress hepatocellular carcinoma progression, Hepatology (Baltimore, Md.). 66 (4) (2017) 1151-1164.

[13] I. Legnini, G. Di Timoteo, F. Rossi, M. Morlando, F. Briganti, O. Sthandier, et al., Circ-ZNF609 Is a Circular RNA that Can Be Translated and Functions in Myogenesis, Molecular cell. 66 (1) (2017) 22-37.e29.

[14] Z. Li, C. Huang, C. Bao, L. Chen, M. Lin, X. Wang, et al., Exon-intron circular RNAs regulate transcription in the nucleus, Nature structural & molecular biology. 22 (3) (2015) 256-264.

[15] Y.Z. Song and J.F. Li, Circular RNA hsa_circ_0001564 regulates osteosarcoma proliferation and apoptosis by acting miRNA sponge, Biochemical and biophysical research communications. 495 (3) (2018) 2369-2375.

[16] Y. Yang, X. Fan, M. Mao, X. Song, P. Wu, Y. Zhang, et al., Extensive translation of circular RNAs driven by N(6)-methyladenosine, Cell research. 27 (5) (2017) 626-641.

[17] Y. Zhang, X.O. Zhang, T. Chen, J.F. Xiang, Q.F. Yin, Y.H. Xing, et al., Circular intronic long noncoding RNAs, Molecular cell. 51 (6) (2013) 792-806.

[18] W.R. Jeck, J.A. Sorrentino, K. Wang, M.K. Slevin, C.E. Burd, J. Liu, et al., Circular RNAs are abundant, conserved, and associated with ALU repeats, RNA (New York, N.Y.). 19 (2) (2013) 141-157.

[19] A. Görlach and S. Holdenrieder, Circular RNA maps paving the road to biomarker development?, Journal of molecular medicine (Berlin, Germany). 95 (11) (2017) 1137-1141.

[20] W.Y. Zhou, Z.R. Cai, J. Liu, D.S. Wang, H.Q. Ju and R.H. Xu, Circular RNA: metabolism, functions and interactions with proteins, Molecular cancer. 19 (1) (2020) 172.

[21] K.L. de Mesy Bentley, R. Trombetta, K. Nishitani, S.N. Bello-Irizarry, M. Ninomiya, L. Zhang, et al., Evidence of Staphylococcus Aureus Deformation, Proliferation, and Migration in Canaliculi of Live Cortical Bone in Murine Models of Osteomyelitis, Journal of bone and mineral research : the official journal of the American Society for Bone and Mineral Research. 32 (5) (2017) 985-990.

[22] S.S. Reilly, M.C. Hudson, J.F. Kellam and W.K. Ramp, In vivo internalization of Staphylococcus aureus by embryonic chick osteoblasts, Bone. 26 (1) (2000) 63-70.

[23] Y. Ren, T. Xue, J. Rainbolt, K.L.M. Bentley, C.A. Galloway, Y. Liu, et al., Efficacy of Bisphosphonate-Conjugated Sitafloxacin in a Murine Model of S. aureus Osteomyelitis: Evidence of "Target & Release" Kinetics and Killing of Bacteria Within Canaliculi, Frontiers in cellular and infection microbiology. 12 (2022) 910970.

[24] E. Adjei-Sowah, Y. Peng, J. Weeks, J.H. Jonason, K.L. de Mesy Bentley, E. Masters, et al., Development of Bisphosphonate-Conjugated Antibiotics to Overcome Pharmacodynamic Limitations of Local Therapy: Initial Results with Carbamate Linked Sitafloxacin and Tedizolid, Antibiotics (Basel, Switzerland). 10 (6) (2021)

[25] M. Jevon, C. Guo, B. Ma, N. Mordan, S.P. Nair, M. Harris, et al., Mechanisms of internalization of Staphylococcus aureus by cultured human osteoblasts, Infection and immunity. 67 (5) (1999) 2677-2681.

[26] S. Pils, T. Schmitter, F. Neske and C.R. Hauck, Quantification of bacterial invasion into adherent cells by flow cytometry, Journal of microbiological methods. 65 (2) (2006) 301-310.

[27] J. Yu, W. Shen, J. Xu, B. Gong, B. Gao and J. Zhu, circUSP42 Is Downregulated in Triple-Negative Breast Cancer and Associated With Poor Prognosis, Technology in cancer research & treatment. 19 (2020) 1533033820950827.

[28] S. Ni, T. Jiang, S. Hao, P. Luo, P. Wang, Y. Almatari, et al., circRNA expression pattern and ceRNA network in the pathogenesis of aseptic loosening after total hip arthroplasty, International journal of medical sciences. 18 (3) (2021) 768-777.

[29] J. Huang, J. Chen and Q. Huang, The profile analysis of circular RNAs in cervical cancer, Medicine. 100 (39) (2021) e27404.

[30] C. Dou, Z. Cao, B. Yang, N. Ding, T. Hou, F. Luo, et al., Changing expression profiles of lncRNAs, mRNAs, circRNAs and miRNAs during osteoclastogenesis, Scientific reports. 6 (2016) 21499.

[31] Z. Sun, X. Cao, Z. Hu, L. Zhang, H. Wang, H. Zhou, et al., MiR-103 inhibits osteoblast proliferation mainly through suppressing Cav1.2 expression in simulated microgravity, Bone. 76 (2015) 121-128.

[32] A. Nicolas, M. Deplanche, P.H. Commere, A. Diot, C. Genthon, W. Marques da Silva, et al., Transcriptome Architecture of Osteoblastic Cells Infected With Staphylococcus aureus Reveals Strong Inflammatory Responses and Signatures of Metabolic and Epigenetic Dysregulation, Frontiers in cellular and infection microbiology. 12 (2022) 854242.

**Figure Legends**

**Figure 1. The intracellular invasion rate by *S. aureus* of osteoblasts infected for 2 h at different MOI.**

A: The invasion rate of *S. aureus* into osteoblasts; B: The average CFSE fluorescence intensity of osteoblasts infected by *S. aureus.*

**Figure 2. The intracellular invasion rate by *S. aureus* on infection for different times at MOI 250.**

A: The invasion rate of *S. aureus* into osteoblasts; B: The average CFSE fluorescence intensity of osteoblasts infected by *S. aureus.*

**Figure 3. The length of identified circRNAs.**

**Figure 4.** **The distributions of identified circRNAs on different chromosomes.**

**Figure 5.** **Classification of identified circRNAs based on the genomic origin.**

**Figure 6. RT-qPCR verification of the DEcircRNAs.**

A: Three upregulated circRNAs; B: Three downregulated circRNAs

**Figure 7. The GO annotation of DEcircRNAs.**

A: The top ten biological processes of upregulated DEcircRNAs; B: The top ten biological processes of downregulated DEcircRNAs; C: The top ten cellular components of upregulated DEcircRNAs; D: The top ten cellular components of downregulated DEcircRNAs; E: The top ten molecular functions of upregulated DEcircRNAs; F: The top ten cellular components of downregulated DEcircRNAs.

**Figure 8. The KEGG pathway enrichment analysis of DEcircRNAs.**

A: The top ten pathways for upregulated DEcircRNAs; B: The top ten pathways for downregulated DEcircRNAs.

**Figure 9.** **The circRNA-miRNA interaction gene network.**

**Table 1 The top ten upregulated circRNAs**

| **CIrcRNA** | **P-value** | **LogFC** | **Chrom** | **Type** | **Source_Gene name** |
| --- | --- | --- | --- | --- | --- |
| hsa_circ_0085769 | 0.006619465 | 5.419799512 | chr8 | exonic | PKT-2 |
| hsa_circ_0004422 | 0.010509051 | 5.257841217 | chr17 | exonic | KANSL1 |
| hsa_circ_0027836 | 0.010902727 | 5.244490038 | chr12 | exonic | APAF1 |
| hsa_circ_0001777 | 0.011372502 | 5.228450418 | chr7 | exonic | ESYT2 |
| hsa_circ_0126072 | 0.016504378 | 5.081633679 | chr4 | exonic | SEL1L3 |
| hsa_circ_0093964 | 0.016504378 | 5.081633679 | chr10 | exonic | DNA2 |
| hsa_circ_0099480 | 0.016504378 | 5.081633679 | chr12 | exonic | EEA1 |
| hsa_circ_0006809 | 0.016504378 | 5.081633679 | chr14 | exonic | CDC42BPB |
| hsa_circ_0118871 | 0.016577017 | 5.078195332 | chr2 | exonic | PARD3B |
| hsa_circ_0102270 | 0.01885345 | 5.023074174 | chr14 | exonic | RTN1 |

**Table 2 The top ten downregulated circRNAs**

| **CircRNA** | **P-value** | **LogFC** | **Chrom** | **Type** | **Source_Gene name** |
| --- | --- | --- | --- | --- | --- |
| hsa_circ_0005232 | 0.001076142 | -5.896583814 | chr2 | exonic | SLC8A1 |
| hsa_circ_0007905 | 0.003607649 | -5.584074167 | chr1 | exonic | STX6 |
| hsa_circ_0002457 | 0.005417129 | -5.456578392 | chr12 | exonic | ATXN2 |
| hsa_circ_0001726 | 0.006080089 | -5.420812827 | chr7 | intronic | ZNF394 |
| hsa_circ_0005332 | 0.006989446 | -5.37673827 | chr3 | exonic | ZBTB20 |
| hsa_circ_0004191 | 0.009407276 | -5.27481415 | chr2 | intronic | PGAP1 |
| hsa_circ_0126873 | 0.00993634 | -5.254030886 | chr4 | exonic | RUFY3 |
| hsa_circ_0005881 | 0.012638753 | -5.163517385 | chr4 | exonic | WHSC1 |
| hsa_circ_0002483 | 0.014426211 | -5.115095648 | chr8 | exonic | PKT-2 |
| hsa_circ_0043244 | 0.016268519 | -5.066004888 | chr17 | exonic | ACACA |

**Table S1 Primers for verification of circRNA expression**

| **Gene name** | **Primer** | **Sequences** |
| --- | --- | --- |
| chr1:213251038-213290752+ | Forward | CCTGCTGAAGCTCACTCAGA |
|  | Reverse | TCGCCCAAACACTATTCCTT |
| chr17:44143903-44172067- | Forward | GCATCGTTCCTCTTTCCAAG |
|  | Reverse | AACATGAGCCTGAAGCCAGT |
| chr8:141856359-141889736- | Forward | GGCGATCATACTGGGAGATG |
|  | Reverse | GAATCCATAGCAGGCCACAT |
| chr2:40655613-40657441- | Forward | GAGGGGAGGATTTTGAGGAC |
|  | Reverse | AAGACTTAATCGCCGCATGT |
| chr1:180953813-180962561- | Forward | GGCTGGACAATGTGATGAAG |
|  | Reverse | GCTAATGCCTGCACAGATGA |
| chr8:141874411-141900868- | Forward | CCCACCAGAGGAGTGGAAAT |
|  | Reverse | AGCAGCTGCCATTATTTTGC |
| GAPDH (internal control) | Forward | GGCCTCCAAGGAGTAAGACC |
|  | Reverse | AGGGGAGATTCAGTGTGGTG |
